# Adenovirus vectors activate Vδ2^+^ γδT cells in a type I interferon-, TNF-, and IL-18-dependent manner

**DOI:** 10.1002/eji.202149367

**Published:** 2022-01-17

**Authors:** Nicholas M. Provine, Ali Amini, Lucy C. Garner, Michael E.B. FitzPatrick, Christina Dold, Laura Silva Reyes, Senthil Chinnakannan, Blanche Oguti, Meriel Raymond, Fulvia Troise, Stefania Capone, Antonella Folgori, Eleanor Barnes, Christine S. Rollier, Andrew J. Pollard, Paul Klenerman

**Affiliations:** 1 Translational Gastroenterology Unit, Nuffield Department of Medicine, University of Oxford, Oxford, UK; 2 Oxford Vaccine Group, Department of Paediatrics, University of Oxford, and the NIHR Oxford Biomedical Research Centre, Oxford, UK; 3 Peter Medawar Building for Pathogen Research, University of Oxford, UK; 4 Nouscom, SRL, Rome, Italy; 5 Ceinge Biotechnologie Avanzate, Naples, Italy; 6 ReiThera, SRL, Rome, Italy

Unlike conventional αβT cells, γδT cells, ~4% of circulating human T cells, are restricted by diverse non-MHC elements [1]. Up to 75% of circulating γδT cells express a paired Vδ2/Vγ9 T cell receptor (TCR) (hereafter Vδ2^+^ γδT cells) [2], which are restricted by butyrophilin 2A and 3A (BTN2A/BTN3A) family proteins, enabling recognition of self- and microbe-derived phosphoantigens [3]. This association of semi-invariant TCR (Vδ2/Vγ9) with monomorphic restricting element (BTN2A/BTN3A) is akin to two other unconventional αβT cell populations: mucosal-associated invariant T (MAIT) cells (Vα7.2-Jα33 TCR binding MR1) and invariant natural killer T (iNKT) cells (Vα24-Jα18/Vβ11 binding CD1d) [4].

Cytokines can activate MAIT and iNKT cells without TCR triggering [4]. Adenovirus (Ad) vaccine vectors, including ChAdOx1, can activate MAIT cells by the cytokines IL-18, type I interferon (IFN), and TNF [5], which activate Vδ2^+^ γδT cells [6],[7],[8]. Thus, we sought to determine if Ad vector-induced cytokines activate Vδ2^+^ γδT cells.

To test this, human peripheral blood mononuclear cells (PBMCs) were stimulated with an increasing multiplicity of infection (MOI) of two Ad vectors: ChAdOx1 or Ad5, and γδT cell activation was measured. After 24 hours, ChAdOx1 induced expression of CD69 and IFN-γ by Vδ2^+^ γδT cells, which were confirmed to co-express Vγ9 TCR (Fig. 1A-1C; Supplemental Fig. 1A-1B). An MOI of 10^3^ viral particles (vp) induced maximal activation (72% CD69^+^ and 26% IFN-γ^+^), which declined at a higher dose. In contrast, Ad5 induced minimal CD69 or IFN-γ expression. Both vectors modestly induced Granzyme B, while negligible TNF was induced (Supplemental Fig. 1C-1D). Activation was biased to the Vδ2^+^ γδT cell subset, as only CD69 was induced on non-Vδ2 γδT cells (Supplemental Fig. 1E).

Species C Ad vectors induce distinct, weaker antiviral cytokine responses than non-species C vectors [9]. We examined three species C vectors (Ad5, Ad6, and ChAdN13) and five non-species C vectors (Ad24, Ad35, ChAd63, ChAd68, and ChAdOx1), and found that on average, the non-species C vectors induced significantly more Vδ2^+^ γδT cell IFN-γ production (Fig. 1D). Thus, Ad vectors can activate Vδ2^+^ γδT cells in vitro, and viral species differ in their stimulatory capacity.

To test if Ad vectors can activate Vδ2^+^ γδT cells in vivo, we collected PBMCs from human volunteers one day pre- and one day post-immunization with a ChAdOx1 MenB.1 construct. Vaccination significantly increased CD69 expression on circulating Vδ2^+^ γδT cells (Fig. 1E). Thus, Ad vectors activate human Vδ2^+^ γδT cells in vivo.

We next determined how Vδ2^+^ γδT cells were activated. Ad vector-driven activation of MAIT cells involves two processes: (1) production of IL-18 by monocytes, and (2) production of type I interferon by plasmacytoid dendritic cells (pDCs), which acts directly but also by triggering TNF production by monocytes [5]. We tested Vδ2^+^ γδT cell activation by these pathways.

Activation of Vδ2^+^ γδT cells by ChAdOx1 was TCR-independent, as blocking BTN3A [10] had no effect (Fig. 2A). Monocyte depletion (Fig. 2B) and IL-18 signaling blockade (Fig. 2C) significantly inhibited Vδ2^+^ γδT cell IFN-γ production. pDC depletion reduced Vδ2^+^ γδT cell IFN-γ production by 71% (*p*=0.06; Fig. 2D). Type I interferon signaling blockade using the vaccinia virus-derived B18R protein significantly reduced Vδ2^+^ γδT cell IFN-γ production (Fig. 2E). TNF signaling blockade using adalimumab (anti-TNF antibody) or etanercept (TNFR2-Fc fusion protein), significantly reduced Vδ2^+^ γδT cell IFN-γ production (Fig. 2F), while a control anti-α4β7 integrin antibody (vedolizumab) had no impact. TNF blockade of PBMCs stimulated with recombinant IFN-α and IL-18 significantly reduced Vδ2^+^ γδT cell IFN-γ production (Fig. 2G), confirming that TNF was an intermediate of type I interferon-induced activation.

We found that Ad vectors activate human Vδ2^+^ γδT cells in vitro and in vivo, and vectors derived from different adenovirus species are differentially stimulatory. Activation involves monocytes and pDCs, and signaling via IL-18, type I interferon, and TNF. This is concordant with the mechanism of Ad vector-induced MAIT cell activation [5], and supports the growing literature of shared innate-like functionality of Vδ2^+^ γδT, MAIT, and iNKT cells [6]–[8].

Our data raise a major question: what contribution do Vδ2^+^ γδT cells make to the immune response induced against the Ad vector-encoded transgene? In mice, which lack an equivalent butyrophilin-restricted γδT cell population [1], the absence of MAIT cells dampens immune responses against the encoded transgene antigen [5]. From the highly overlapping biology of MAIT cells and Vδ2^+^ γδT cells in humans, we hypothesize that these populations have possibly redundant roles in modulating vaccine immunogenicity. Further studies will be required to disentangle the shared functionality of these populations.

## Supplementary Material

Supplementary data

## Figures and Tables

**Figure 1:**
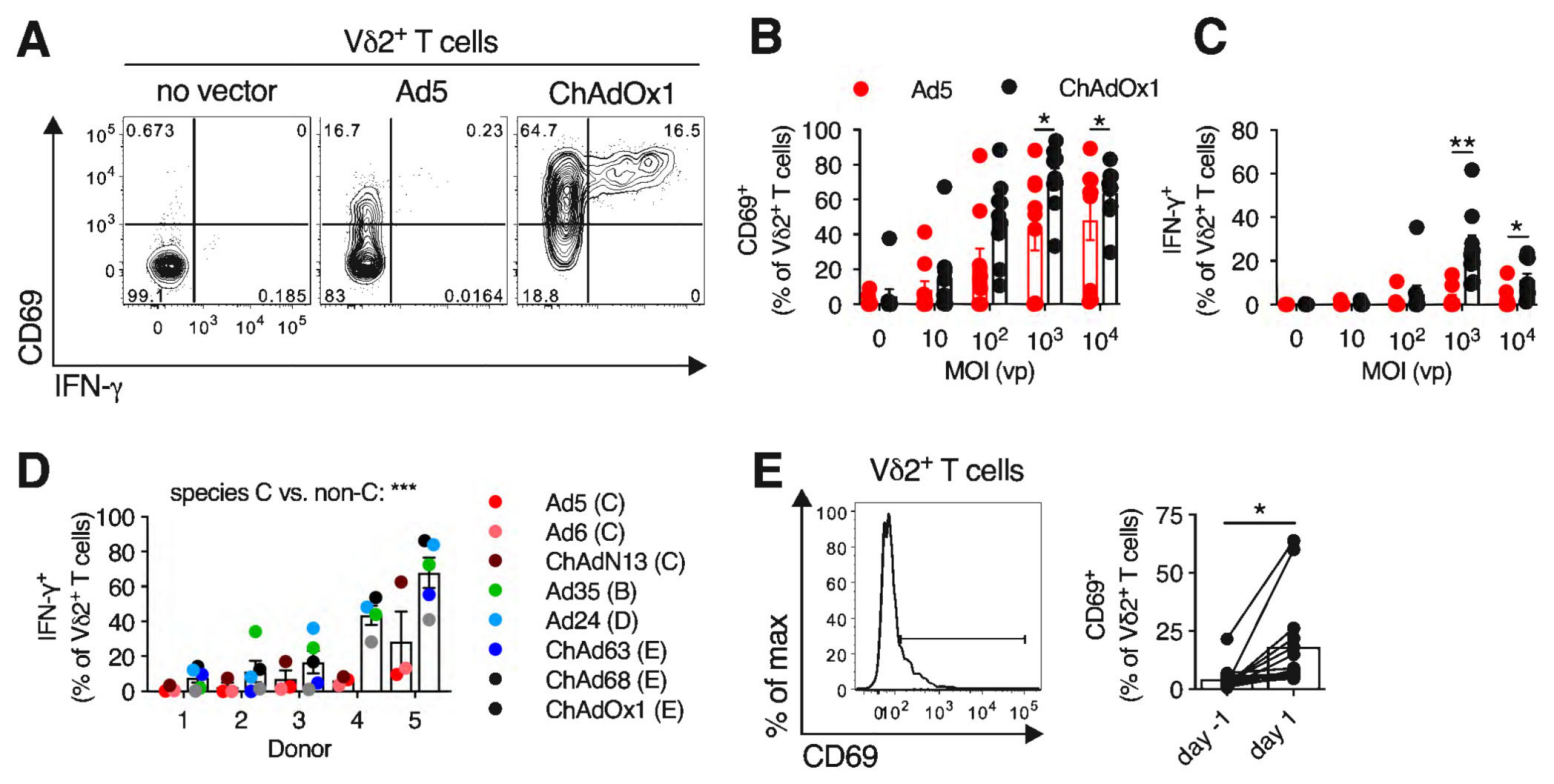
Adenovirus vectors activate Vδ2^+^ γδT cells in vitro and in vivo. (A-C) Human PBMCs were stimulated with an increasing MOI of Ad5-GFP or ChAdOx1-GFP for 24 hours. (A) Representative Vδ2^+^ γδT cell CD69 and IFN-γ expression [MOI=10^3^ vp (viral particles)]. (B,C) Summary (*n*=9 donors, three experiments) of Vδ2^+^ γδT cell CD69 (B) and IFN-γ (C) expression. (D) Human PBMCs were stimulated with the indicated vectors (viral species in parentheses; *n*=4 donors for ChAd63 and *n*=5 donors for all others, two experiments). Vδ2^+^ γδT cell IFN-γ expression was measured after 24 hours. (E) PBMCs from human volunteers (*n*=14 volunteers, two experiments) were collected one day before and one day after vaccination with ChAdOx1 MenB.1 (5×10^10^ vp dose) and Vδ2^+^ γδT cell CD69 expression was measured. Mean ± SEM are shown. Dots represent individual donors/volunteers. *p < 0.05, **p < 0.01, ***p < 0.001. (B,C) Unpaired *t* test; (D) Two-way ANOVA; (E) Paired Wilcoxon rank-sum test.

**Figure 2:**
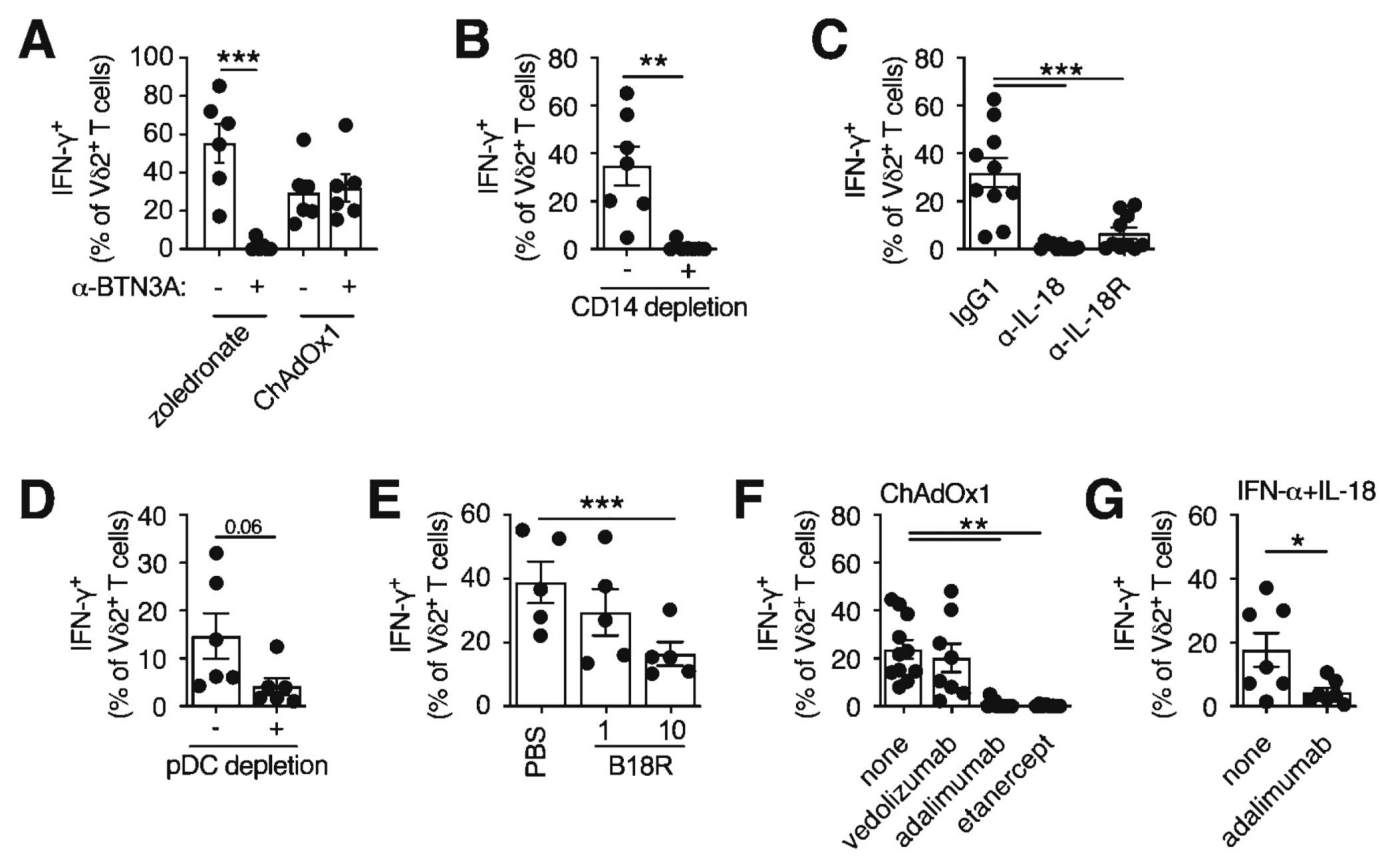
Activation of Vδ2^+^ γδT cells by ChAdOx1 requires IL-18, type I interferon, and TNF. In all experiments, Vδ2^+^ γδT cell IFN-γ production was measured after 24 hours. (A) PBMCs (*n=*6 donors, two experiments) were stimulated with zoledronate or ChAdOx1-GFP plus anti-BTN3A blocking or control antibody. (B) PBMCs or CD14-depleted PBMCs (*n*=7 donors, two experiments) were stimulated with ChAdOx1-GFP. (C) PBMCs (*n*=10 donors, three experiments) were treated with anti-IL-18 or anti-IL-18Rα antibodies (10 μg/ml) and stimulated with ChAdOx1-GFP. (D) PBMCs or CD123-depleted PBMCs (*n*=6 donors, two experiments) were stimulated with ChAdOx1-GFP. (E) PBMCs (*n*=5 donors, two experiments) were treated with B18R (type I interferon antagonist; 1 or 10 μg/ml) and stimulated with ChAdOx1-GFP. (F) PBMCs (three experiments) were treated with vedolizumab (anti-α4β7 integrin antibody; *n*=8 donors), adalimumab (anti-TNF antibody; *n=*11 donors), or etanercept (TNFR2-Fc fusion protein; *n=*8 donors), or untreated (*n=*11 donors), and stimulated with ChAdOx1-GFP. (G) PBMCs (*n*=7 donors, two experiments) were treated with adalimumab (10 μg/ml) and stimulated with recombinant IFN-α and IL-18. Mean ± SEM are shown. Dots represent individual donors. *p < 0.05, **p < 0.01, ***p < 0.001. (A,B,D,G) Unpaired *t* test; (C) Repeated-measure one-way ANOVA with Holm-Sidak’s multiple comparison test; (E) Repeated-measure one-way ANOVA with test for linear trend; (F) Mixed-effects analysis with Dunnett’s multiple comparison test.

## Data Availability

The data that support the findings of this study are available from the corresponding authors upon reasonable request.
