# Supplementary material for: Adenovirus vectors activate Vδ2+ γδT cells in a type I interferon-, TNF-, and IL-18-dependent manner
Source: Eur J Immunol. Author manuscript; Available in PMC 2026 Aug 11. (PMC7619350; doi:10.1002/eji.202149367)
Supplement: Supplementary file 1 [file EMS217504-supplement-Supplementary_data.pdf]

Supporting Information for:

Adenovirus vectors activate Vδ2^{+} γδT cells in a type I interferon-, TNF-, and IL-18-dependent manner

Nicholas M. Provine[1,*,#], Ali Amini[1,*], Lucy C. Garner[1], Michael E.B. FitzPatrick[1], Christina Dold[2], Laura Silva Reyes[2], Senthil Chinnakannan[3], Blanche Oguti[2], Meriel Raymond[2], Fulvia Troise[4,5], Stefania Capone[6], Antonella Folgori[6], Eleanor Barnes[1,3], Christine S. Rollier[2,7], Andrew J. Pollard[2], Paul Klenerman[1,3,#]

[1] Translational Gastroenterology Unit, Nuffield Department of Medicine, University of Oxford, Oxford, UK

[2] Oxford Vaccine Group, Department of Paediatrics, University of Oxford, and the NIHR Oxford Biomedical Research Centre, Oxford, UK

[3] Peter Medawar Building for Pathogen Research, University of Oxford, UK

[4] Nouscom, SRL, Rome, Italy

[5] Ceinge Biotechnologie Avanzate, Naples, Italy

[6] ReiThera, SRL, Rome, Italy

[7] Current affiliation: School of Biosciences and Medicine, University of Surrey, Guildford, UK

* contributed equally

[#] Corresponding authors: nicholas.provine@ndm.ox.ac.uk; paul.klenerman@ndm.ox.ac.uk

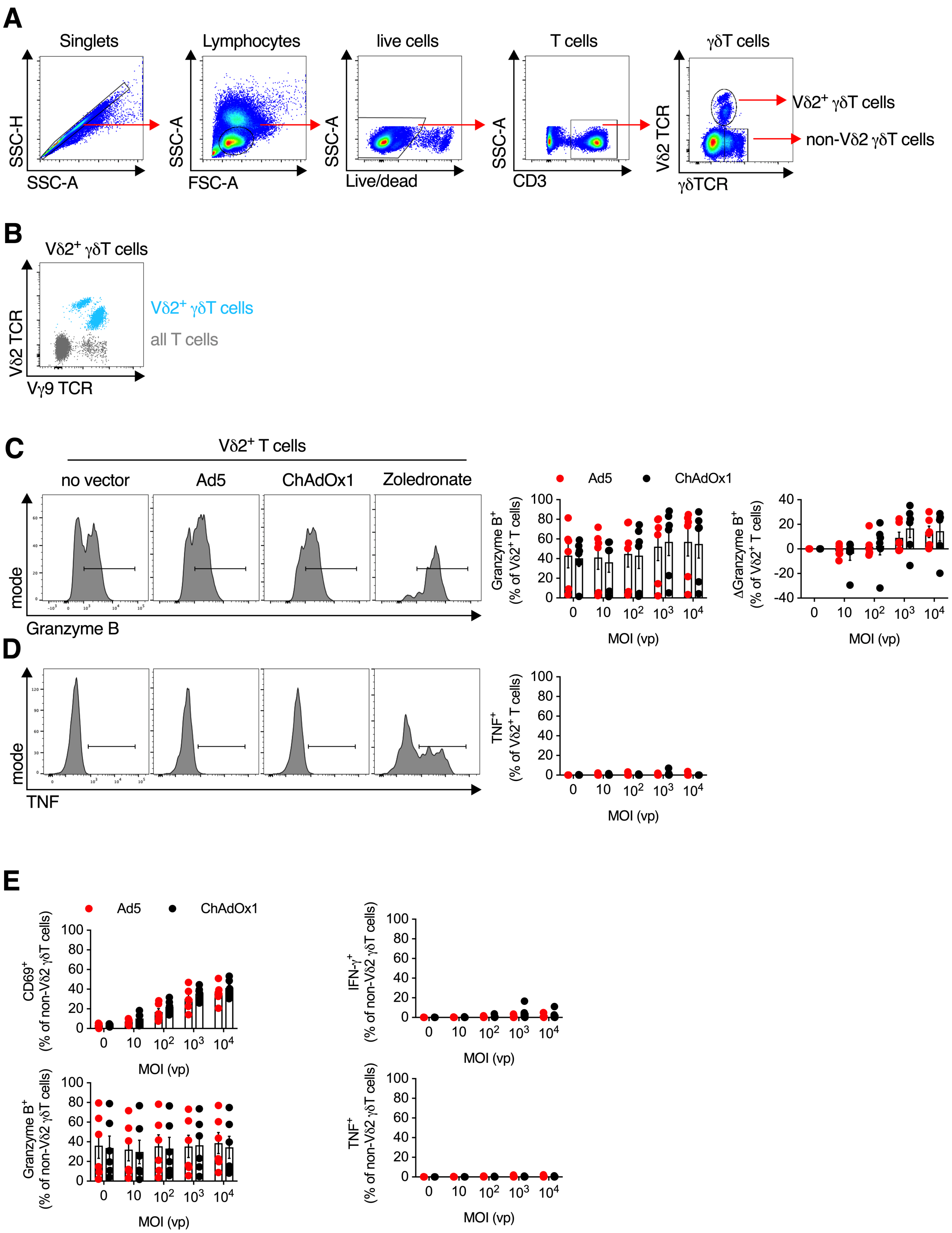

Supplemental Figure 1. (A) Gating scheme to identify Vδ2⁺ γδT cells and non-Vδ2 γδT cells in human PBMCs. (B) Representative staining (*n*=6, two experiments) of Vγ9 TCR on Vδ2⁺ γδT cells. (C-E) Human PBMCs (*n*=6 donors, two experiments) were stimulated with an increasing

MOI of Ad5-GFP or ChAdOx1-GFP. (C,D) Representative plots and group summary for Vδ2$^+$ γδT cell Granzyme B (C) and TNF (D) expression measured after 24 hours. (C, *right panel*) Vδ2$^+$ γδT cell Granzyme B expression normalized to per-donor expression at MOI=0. (E) Group summary for CD69, IFN-γ, Granzyme B, and TNF expression on non-Vδ2 γδT cells after 24 hours.

Materials and methods

Viral vectors

The following E1/E3-deleted adenovirus vectors were produced in HEK293 cells [1] by the Jenner Institute Viral Vector Core Facility (University of Oxford): Ad5-GFP, ChAdOx1-GFP, and ChAd63-GFP. The Clinical Biomanufacturing Facility (University of Oxford) produced the ChAdOx1 MenB.1 construct used for testing in human volunteers in PER.C6 cells [2]. Nouscom, SRL (Rome, Italy) produced the E1/E3-deleted ChAdN13 construct, and Reithera, SRL (Rome, Italy) produced the E1/E3-deleted Ad6, Ad24, Ad35, and ChAd68 constructs in HEK293 cells [3]. All vectors were isolated and purified by $CsCl_2$ ultracentrifugation.

Human PBMC isolation

Human peripheral blood mononuclear cells (PBMCs) were isolated as previously described [4]. Samples were collected from healthy blood donors either as anonymized samples from the NHS Blood and Transplant Service or through the "Gastrointestinal Illness in Oxford: prospective cohort for outcomes, treatment, predictors and biobanking" (Ref: 16/YH/0247). Written informed consent was received from all donors.

Measurement of Vδ2$^+$ $\gamma\delta$T activation in vaccinated human volunteers

Healthy volunteers aged 18-50 were enrolled in the clinical trial ISRCTN trial number: ISRCTN46336916. Written informed consent was obtained from all participants. PBMCs were collected one day before and one day after a boosting immunization with ChAdOx1 MenB.1 at a dose of 5×10^{10} vp and cryopreserved (10% DMSO/90% FBS) until analysis.

Cryopreserved PBMCs were thawed rapidly at 37°C and transferred to pre-warmed R10 media (RPMI-1640 + 10% FBS + 1 % Penicillin/Streptomycin). Cells were washed once and then 2×10^6 cells added per well to a 96-well U-bottom plate. Cells were washed a further time with FACS buffer (PBS + 0.05% BSA + 1% EDTA). Surface staining was for 30 min at 4°C with: anti-CD19 (clone HIB19, 1 in 100), anti-CD14 (clone M5E2, 1 in 100), anti-CD3 (clone UCHT1, 1 in 100), anti-Vδ2 TCR (clone B6, 1 in 100), and anti-CD69 (clone FN50, 1 in 100). Cells were washed two times in FACS buffer and then stored at 4°C until sample acquisition on a BD Aria III (BD Biosciences). SYTOX Green viability dye (1 in 6000) was added prior to sample acquisition.

In vitro stimulation of human PBMCs

For in vitro stimulation with Ad vectors, fresh PBMCs were used. For cytokine stimulation of PBMCs, cryopreserved PBMCs were used, and thawed as described above. $CD14^+$ monocyte depletion was performed using CD14 MicroBeads and plasmacytoid dendritic cells (pDCs) were depleted using CD123 MicroBeads (both Miltenyi Biotec), per the manufacturer's instructions. One million cells were added to a 96-well U-bottom plate.

Unless indicated otherwise, Ad vectors were added at an MOI (multiplicity of infection) of 10^3 vp (viral particles). IFN-α2a (Sigma-Aldrich) and IL-18 (R&D systems) were each added at a final concentration of 50 ng/ml. Zoledronate (Sigma-Aldrich) was added at a final concentration of 45 µM. Stimulation was for 24 hours at 37°C in 5% CO_2. Brefeldin A (1 in 1000; BioLegend) was added after 20 hours of stimulation.

Blocking antibodies were added immediately prior to addition of vectors/cytokines. Anti-Butyrophilin 3A (BTN3A; clone 103.2; kind gift of ImCheck Therapeutics), anti-IL-18 antibody (clone 125-2H; R&D Systems), anti-IL-18Rα antibody (clone 70625; R&D Systems), vedolizumab (anti-α4β7 integrin antibody; Takeda Pharmaceuticals), adalimumab (anti-TNF antibody; AbbVie Inc), and etanercept (TNFR2-Fc fusion protein; kind gift of Dr. Hussein Al-Mossawi, University of Oxford) were all used at a final concentration of 10 µg/ml. The vaccinia virus-derived type I interferon antagonist B18R (Thermo Fisher Scientific) was used at a final concentration of either 1 or 10 µg/ml.

Following stimulation, cells were washed once with FACS buffer. Cells were stained with fixable viability dye (1 in 400; Life Technologies) for 15 min at 4°C. Cells were washed twice with FACS buffer, and incubated for 20 min at 4°C with Cytofix/Cytoperm solution (BD Biosciences). Following two washes with Perm/Wash buffer (BD Biosciences), samples were stained in Perm/Wash buffer with the following: anti-CD3 (clone OKT3 or UCHT1, 1 in 100), anti-γδTCR (clone B1, 1 in 50), anti-Vδ2 TCR (clone B6, 1 in 100), anti-Vγ9 TCR (clone B3, 1 in 100), anti-CD69 (clone FN50, 1 in 100), anti-IFN-γ (clone B27, 1 in 100), anti-TNF (clone MAb11, 1 in 100), and anti-Granzyme B (clone GB11, 1 in 100). After 30 min at 4°C, samples were washed two times with Perm/Wash buffer, resuspended in FACS buffer, and stored at 4°C

until data acquisition. All samples were acquired on a BD Fortessa flow cytometer or BD LSR II flow cytometer (BD Biosciences).

Data analysis and statistics

All data was analyzed in FlowJo v. 9.9.6 (FlowJo, LLC). Statistical analysis and graphs were generated using Prism v. 8.4.3 (GraphPad). The statistical test used for analysis is indicated in the figure legend. For statistical testing, $p<0.05$ was considered significant, and $p<0.1$ was considered a trend.

References

1. Dicks MDJ, Kremer EJ, Spencer AJ, Edwards NJ, Wadell G, Bojang K, Gilbert SC, *et al.* A Novel Chimpanzee Adenovirus Vector with Low Human Seroprevalence: Improved Systems for Vector Derivation and Comparative Immunogenicity. *PLoS ONE*. 2012; 7:e40385. Available at: http://dx.plos.org/10.1371/journal.pone.0040385.s001.DOI: 10.1371/journal.pone.0040385.s001.

2. Antrobus RD, Coughlan L, Berthoud TK, Dicks MD, Hill AV, Lambe T, Gilbert SC. Clinical Assessment of a Novel Recombinant Simian Adenovirus ChAdOx1 as a Vectored Vaccine Expressing Conserved Influenza A Antigens. *Molecular therapy*. 2013; 22:668–674. Available at: http://www.nature.com/doifinder/10.1038/mt.2013.284.DOI: 10.1038/mt.2013.284.

3. Colloca S, Barnes E, Folgori A, Ammendola V, Capone S, Cirillo A, Siani L, *et al.* Vaccine vectors derived from a large collection of simian adenoviruses induce potent cellular immunity across multiple species. *Science Translational Medicine*. 2012; 4:115ra2. Available at: http://stm.sciencemag.org/cgi/doi/10.1126/scitranslmed.3002925.DOI: 10.1126/scitranslmed.3002925.

4. Provine NM, Binder B, FitzPatrick MEB, Schuch A, Garner LC, Williamson KD, Wilgenburg B van, *et al.* Unique and Common Features of Innate-Like Human Vδ2+ γδT Cells and Mucosal-Associated Invariant T Cells. *Frontiers in Immunology*. 2018; 9:756. Available at: http://eutils.ncbi.nlm.nih.gov/entrez/eutils/elink.fcgi?dbfrom=pubmed&id=29740432&retmode=ref&cmd=prlinks.DOI: 10.3389/fimmu.2018.00756.
